# SiC Double-Trench MOSFETs with an Integrated MOS-Channel Diode for Improved Third-Quadrant Performance

**DOI:** 10.3390/mi16030244

**Published:** 2025-02-20

**Authors:** Zhiyu Wang, Hongshen Wang, Yuanjie Zhou, Qian Liu, Hao Wu, Jian Shen, Juan Luo, Shengdong Hu

**Affiliations:** 1 School of Microelectronics and Communication Engineering, Chongqing University, Chongqing 400030, China; 2 National Key Laboratory of Integrated Circuits and Microsystems, Chongqing 401332, China

**Keywords:** drain-induced barrier-lowering effect, MOS-channel diode (MCD), double-trench MOSFET, SiC MOSFET

## Abstract

In this article, a novel double-trench SiC MOSFET with an integrated MOS-channel diode (MCD) is proposed and analyzed through TCAD simulations. The MCD incorporates a short channel, where the channel length can be adjusted by modifying the recess depth. Owing to the drain-induced barrier-lowering (DIBL) effect, a low potential barrier is created for electrons flowing from the JFET region to the N+ source region. This effectively eliminates the bipolar degradation of the parasitic body p-i-n diode and reduces the cut-in voltage $V_{\mathrm{on}}$ by 69.2%. Additionally, the breakdown voltage (BV) remains nearly unchanged. The reduction in the p-well region alleviates the JFET effect, successfully lowering the specific on-resistance $R_{\mathrm{on},\mathrm{sp}}$, making the channel easier to turn on, and reducing the threshold voltage ($V_{\mathrm{th}}$). However, the increase in the gate charge $Q_{g}$ results in a slight rise in the switching loss.

## 1. Introduction

Silicon carbide (SiC) is distinguished by its superior material properties, including a higher breakdown electric field strength and a larger bandgap, which enable the development of devices with ultra-low on-state resistance and outstanding switching characteristics. These advantages make SiC one of the most attractive wide-bandgap semiconductor materials for power electronics applications [1,2]. Additionally, these properties make SiC particularly well suited for applications that require high power density and efficiency in power conversion [3,4]. In many applications, the body diode of a MOSFET plays a crucial role by functioning as a freewheeling diode, as is commonly seen in voltage source inverters [5]. In most power converter circuits, the output power is regulated by the alternating conduction of the switch and the freewheeling diode, which, in turn, carries the load current. Due to the cost advantages of the system, the prospect of using the intrinsic body diode of the MOSFET as the freewheeling diode in these applications is particularly attractive [6]. However, the parasitic body diode of the SiC MOSFET is unsuitable for use in many applications for two reasons. First, the wide bandgap of SiC results in a high turn-on voltage ($V_{\mathrm{on}}$) for its body diode, leading to increased switching loss [7,8]. Second, bipolar degradation, a widely acknowledged issue, is caused by the formation of stacking faults (SFs) and has an adverse effect on long-term reliability [9,10].

To address the aforementioned problems, SiC MOSFETs with embedded unipolar diodes have been proposed. These designs enhance reverse conduction performance by achieving a low $V_{\mathrm{on}}$ while effectively eliminating bipolar degradation. One notable approach involves integrating junction barrier Schottky (JBS) diodes or Schottky barrier diodes (SBD) into SiC MOSFETs, as demonstrated in several studies [11,12,13,14,15,16,17]. However, JBS and SBD diodes exhibit much higher leakage currents, particularly under high-temperature operating conditions [18,19]. A SiC MOSFET with an integrated heterojunction diode has been proposed and studied as a potential alternative [20,21,22,23]. However, this approach entails a complex and challenging manufacturing process and may lead to an increased electric field at the heterojunction interface [24]. The integration of the normally OFF JFET diode with the SiC MOSFET has also been proposed and investigated [25,26]. However, the electrical characteristics of the normally OFF JFET diode are quite sensitive to the mesa width [27]. An SiC MOSFET with an integrated MOS-channel diode (MCD) has also been proposed [28]. However, due to the presence of oxide on the bottom trench surface, the P+ region at the bottom of the source-contact trench remains floating.

In this article, a double-trench MOSFET (DTMOS) with an integrated MOS-channel diode (MCD), which improves reverse conduction performance, is proposed and demonstrated through TCAD simulations. Compared to the conventional SiC DTMOS, the proposed structure eliminates bipolar degradation and exhibits superior reverse conduction performance with almost no impact on other device characteristics.

## 2. Device Structure and Mechanism

**Figure 1:**
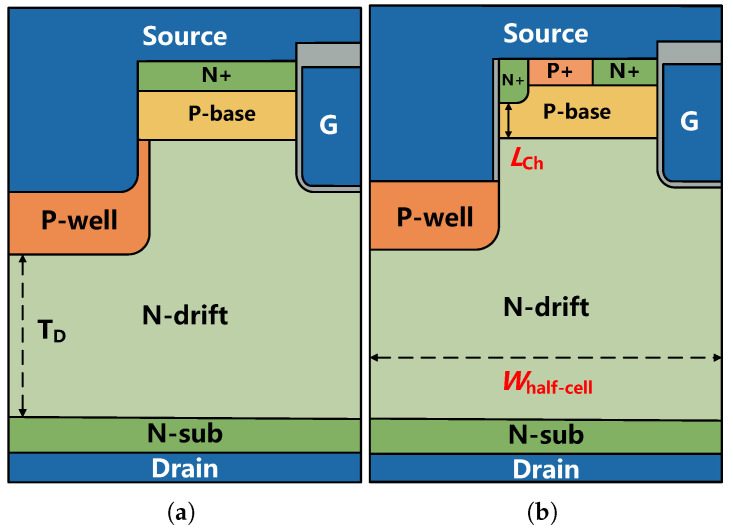
Schematic cross-sections of (**a**) the conventional SiC DTMOS and (**b**) the proposed SiC MOSFET.

The schematic cross-sections of the conventional SiC DTMOS and the proposed MOSFET are shown in Figure 1a,b. In both SiC MOSFETs studied, a grounded P+ shield region under the recessed source is implemented to shield the field near the gate bottom from high electric fields. The key distinction from the previously proposed SiC MOSFET [28] is that the P+ region at the bottom of the source-contact trench is grounded rather than floating, which also simplifies the fabrication process. At the same time, it reduces the JFET effect caused by the addition of a P-well region at the bottom of the gate trench, achieving a good trade-off between breakdown voltage (BV) and $R_{\mathrm{on},\mathrm{sp}}$. Compared to the conventional DTMOS, the proposed MOSFET incorporates an MCD consisting of N+, P-base, and N-drift regions, achieved by forming an oxide layer along the sidewalls of the source trench. The main device parameters used in the simulation are listed in Table 1.

**Figure 2:**
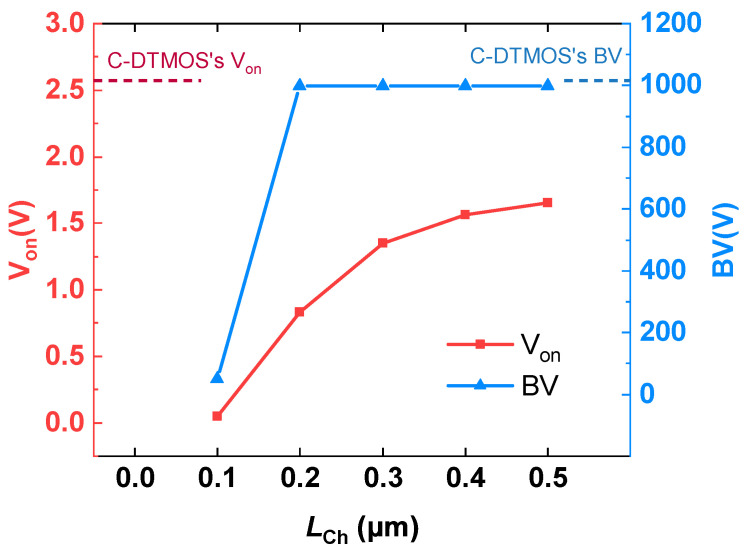
Influence of $L_{\mathrm{Ch}}$ on the cut-in voltage $V_{\mathrm{on}}$ and the breakdown voltage in the proposed SiC MOSFET.

Figure 2 shows the impact of the channel length $L_{\mathrm{Ch}}$ on both the cut-in voltage $V_{\mathrm{on}}$ and the breakdown voltage (BV) in the proposed SiC MOSFET. As shown, the breakdown voltage remains almost constant across various values of $L_{\mathrm{Ch}}$, with only a slight change observed when $L_{\mathrm{Ch}}$ is reduced to 0.2 μm. This indicates that the breakdown voltage is largely unaffected by changes in channel length until it reaches 0.1 μm. On the other hand, the cut-in voltage $V_{\mathrm{on}}$ exhibits a noticeable decrease as $L_{\mathrm{Ch}}$ is reduced. Specifically, when $L_{\mathrm{Ch}}$ is reduced from 0.5 μm to 0.2 μm, $V_{\mathrm{on}}$ decreases from 1.6 V to 0.8 V, representing a 69.2% reduction compared to the conventional structure. Notably, the breakdown voltage (BV) remains almost unchanged, which suggests that the device’s overall voltage withstand capability is preserved even with the reduction in channel length, making this design approach highly efficient for achieving lower cut-in voltage without compromising the breakdown voltage.

**Figure 3:**
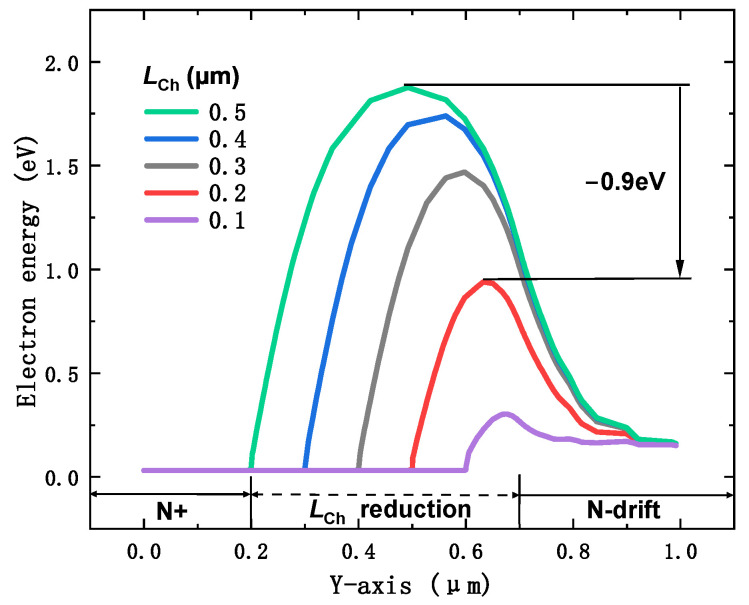
Conduction band energy $E_{C}$ along the SiC/oxide interface with different $L_{\mathrm{Ch}}$ at zero applied bias.

This behavior is attributed to the continuous reduction in the potential barrier, as shown in Figure 3. The electron potential barrier decreases with the reduction in $L_{\mathrm{Ch}}$, a phenomenon driven by the drain-induced barrier-lowering (DIBL) effect, as demonstrated in the experimental works of [30,31]. As the channel length shortens, the barrier that electrons must overcome to flow from the source to the drain is significantly lowered, which, in turn, reduces the cut-in voltage $V_{\mathrm{on}}$. As shown in Figure 3, reducing $L_{\mathrm{Ch}}$ from 0.5 to 0.2 μm decreases the electron potential barrier by 0.9 eV, which, in turn, lowers the $V_{\mathrm{on}}$ of the MOSFET. This reduction allows the proposed SiC MOSFET to achieve a significantly lower cut-in voltage with minimal adjustments to the device structure. Based on this trade-off, $L_{\mathrm{Ch}}=0.2\;$μm is selected for further analysis, as it offers a good balance between the desired reduction in $V_{\mathrm{on}}$ and the stability of the breakdown voltage.

**Figure 4:**
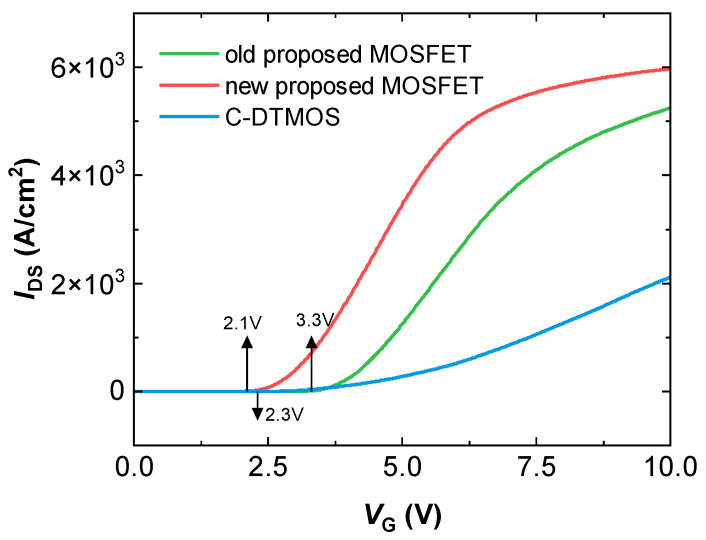
Transfer characteristics of the conventional SiC DTMOS, the previously proposed SiC MOSFET, and the newly proposed SiC MOSFET.

Figure 4 presents the transfer characteristics of the conventional SiC DTMOS, the previously proposed SiC MOSFET, and the newly proposed SiC MOSFET. Although the previous MOSFET offers comparable advantages in electrical performance and reliability to the new MOSFET, its structural differences enable it to exhibit the small mesa effect. As a result, the mesa between the P+ shield regions is relatively narrow, causing the threshold voltage ($V_{\mathrm{th}}$) to increase from 2.1 V to 3.3 V, which is a 57% increase. In addition, compared to the conventional DTMOS, the reduction in the area of the P+ region results in a decrease in $V_{\mathrm{th}}$ from 2.3 V to 2.1 V, which is a reduction of 8.7%.

**Figure 5:**
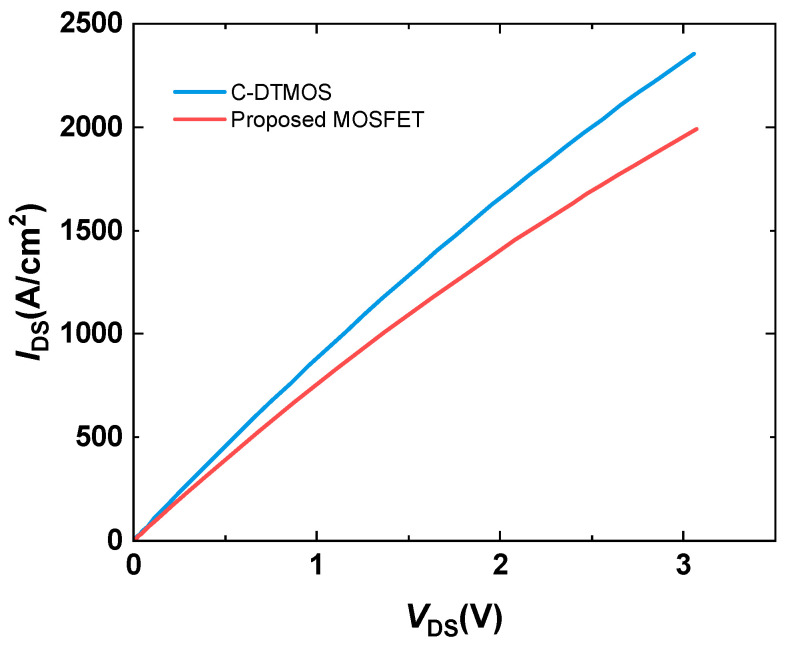
Forward conduction characteristics of the conventional SiC DTMOS and the proposed SiC MOSFET.

Figure 5 shows the forward conduction characteristics of the conventional SiC DTMOS and the proposed SiC MOSFET. From the curves, the $R_{\mathrm{on},\mathrm{sp}}$ values of the conventional DTMOS and the proposed SiC MOSFET are calculated to be 1.427 mΩ∗cm^2^ and 1.209 mΩ∗cm^2^, respectively, indicating a 15% reduction in $R_{\mathrm{on},\mathrm{sp}}$ for the proposed structure. This improvement underscores the enhanced performance of the proposed MOSFET compared to the conventional DTMOS. The reduction in $R_{\mathrm{on},\mathrm{sp}}$ is primarily attributed to the reduction in the P-well region, which effectively alleviates the JFET effect, contributing to lower resistance.

In this study, Sentaurus TCAD tools are used to perform the device simulations and the mixed-mode simulations. The detailed parameters of the cell are provided in Table 1. Several critical models are incorporated [27], including Shockley–Read–Hall recombination, Auger recombination, the Okuto–Crowell model for BV simulations, incomplete ionization, doping-dependent transport, bandgap narrowing, barrier lowering, and anisotropic material properties. Additionally, mobility models considering doping dependence, high-field saturation, and the normal effect are included. Acceptor and donor traps in the P-type region, as well as contact resistance, are included as key factors in suppressing the body diode current during third-quadrant conduction, as reported in [30]. The SiC/SiO_2_ interface trap density is set to $1\times 10^{12}\;{\mathrm{cm}}^{-2}$ [28].

## 3. Simulation Results and Discussion

**Figure 6:**
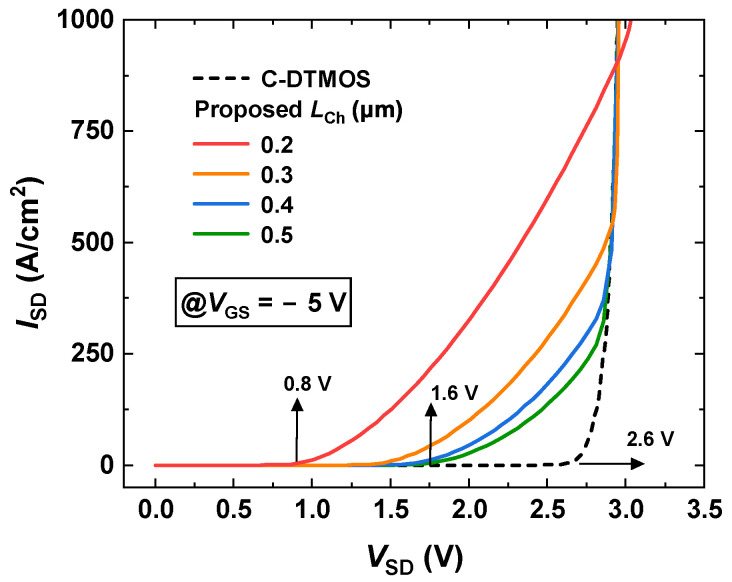
Reverse conduction characteristics of the DTMOS and the proposed SiC MOSFET.

Figure 6 shows the reverse conduction characteristics of both the conventional DTMOS and the proposed SiC MOSFET at $V_{\mathrm{GS}}=-5$ V. In the conventional DTMOS, the body diode was responsible for conducting the reverse current, with a cut-in voltage $V_{\mathrm{on}}$ of 2.6 V ($I_{\mathrm{SD}}\;=\;1\;{A/\mathrm{cm}}^{2}$). In the conventional structure, the source-drain current $I_{\mathrm{SD}}$ was primarily composed of the hole current, which led to bipolar degradation. Bipolar degradation can result in stacking faults and other reliability issues, severely compromising the device’s performance. Therefore, it was crucial to mitigate or eliminate this effect to ensure device reliability and performance. In the proposed MOSFET, when $L_{\mathrm{Ch}}$ was reduced from 0.5 to 0.2 μm, $V_{\mathrm{on}}$ decreased from 1.6 V to 0.8 V. This reduction is attributed to the drain-induced barrier-lowering (DIBL) effect, which is illustrated in Figure 3. Compared to the conventional DTMOS, $V_{\mathrm{on}}$ was reduced by 69.2%. More importantly, in the proposed SiC MOSFET, the conduction current $I_{\mathrm{SD}}$ was carried by electrons instead of holes. This change effectively eliminated the bipolar degradation seen in conventional DTMOS devices. By replacing hole conduction with electron conduction, the proposed MOSFET avoids the reliability issues associated with bipolar degradation, thus ensuring better overall performance, enhanced reliability, and a longer operational lifespan.

**Figure 7:**
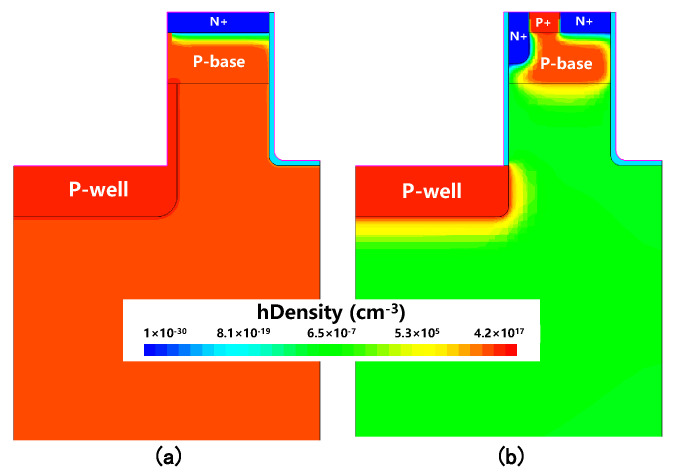
Hole density distributions at $I_{\mathrm{SD}}$ = 100 ${A/\mathrm{cm}}^{2}$ for (**a**) conventional SiC DTMOS and (**b**) proposed MOSFET.

**Figure 8:**
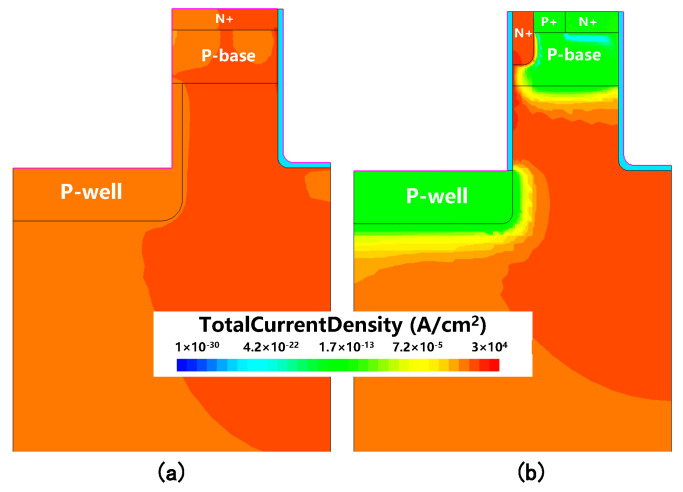
Total current density distributions at $I_{\mathrm{SD}}$ = 100 ${A/\mathrm{cm}}^{2}$ for (**a**) conventional SiC DTMOS and (**b**) proposed MOSFET.

Figure 7a shows the hole current distribution at $I_{\mathrm{SD}}=100\;{A/\mathrm{cm}}^{2}$ in the conventional DTMOS, highlighting the presence of hole current flowing through the P-base region. In contrast, Figure 7b shows the hole current distribution in the proposed MOSFET, where it is evident that no hole current flowed through the P-base region. Further analysis of the total current density distributions at $I_{\mathrm{SD}}=100\;{A/\mathrm{cm}}^{2}$ is presented in Figure 8a,b for the conventional DTMOS and the proposed MOSFET, respectively. From these figures, it is clear that in the proposed SiC MOSFET, the MOS-channel diode was turned on. As a result, all reverse conduction current was carried out by the MOS-channel diode, preventing the flow of current through the body diode. This observation confirms that the body diode in the proposed structure was completely deactivated, effectively eliminating the risk of bipolar degradation. This represents a significant improvement over the traditional DTMOS structure.

**Figure 9:**
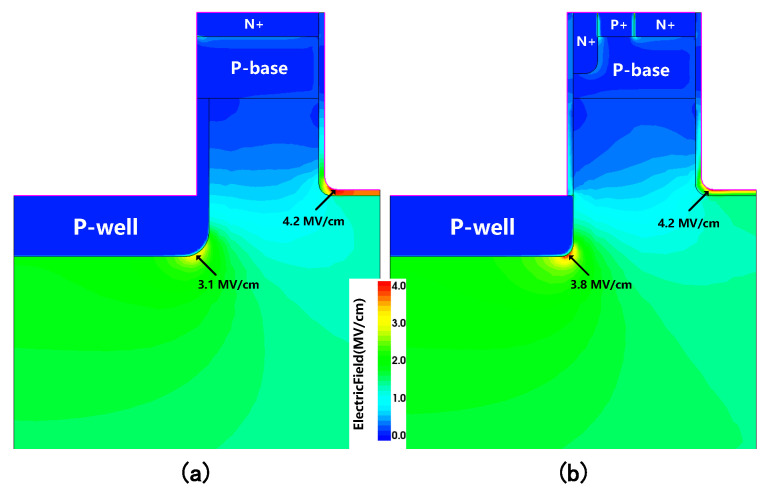
Electric field distributions at $V_{\mathrm{DS}}$ = 700 V of (**a**) conventional SiC DTMOS and (**b**) proposed MOSFET.

The electric field distributions at $V_{\mathrm{DS}}=700\;V$ for the two studied SiC MOSFETs are shown in Figure 9. In MOSFETs, the electric field is typically concentrated near the gate oxide, especially at the corners of the trench, which can lead to significant reliability concerns. This is because, in the rounded corners of the trench, where the curvature is lower, the electric field lines tend to concentrate more densely, resulting in the highest electric field strength in these regions. Consequently, the maximum electric field in each device occurred in the gate oxide at the trench corners. Despite modifications to the source trench and a reduction in the p-well area, the $E_{\mathrm{ox}}$ in the proposed MOSFET showed almost no degradation compared to the conventional DTMOS. Most of the electric field in the gate oxide was below the safety limit (4 MV/cm [8]), ensuring the long-term reliability of the gate oxide.

**Figure 10:**
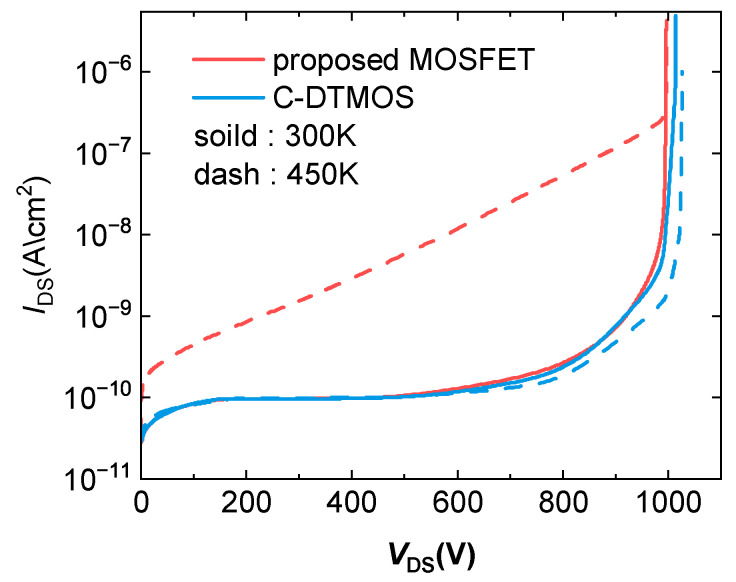
Blocking characteristics of conventional SiC DTMOS and proposed SiC MOSFET.

Figure 10 illustrates the behavior of the conventional SiC DTMOS and the proposed SiC MOSFET at different temperatures. At 300 K, the breakdown voltage of the proposed structure was nearly identical to that of the conventional structure. However, when the temperature rose to 450 K, the leakage current in the proposed structure increased significantly due to a reduction in the potential barrier. Despite the increase in the leakage current, the breakdown voltage of the proposed structure remained almost the same as it was at 300 K.

**Figure 11:**
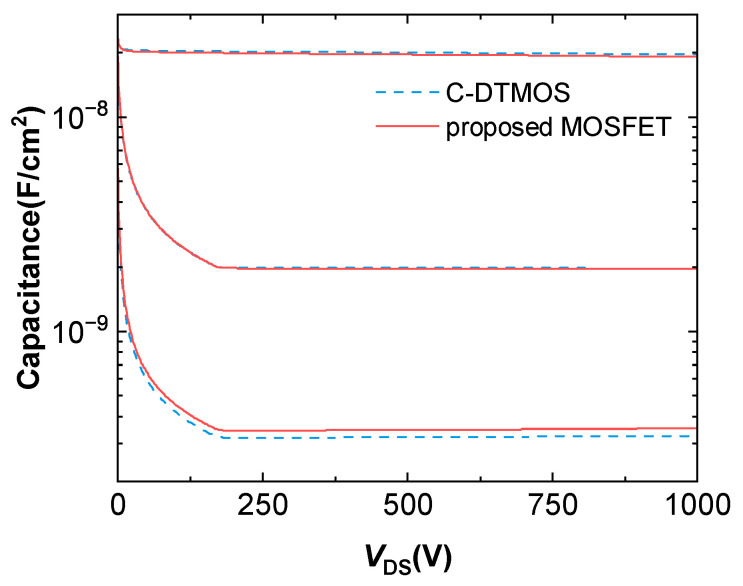
C–V characteristics of the conventional SiC DTMOS and the proposed MOSFET.

**Figure 12:**
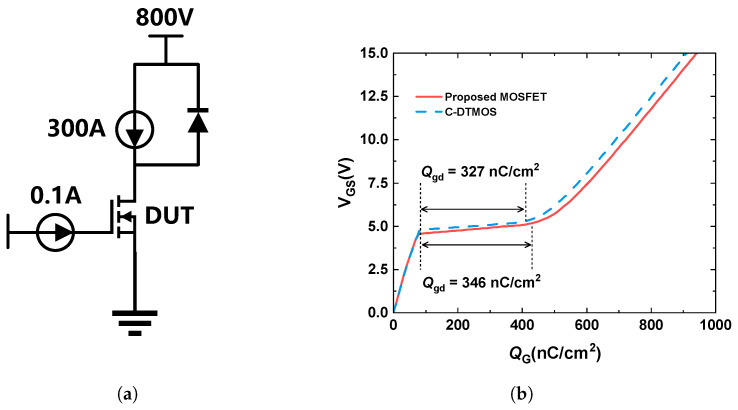
(**a**) Test circuit. (**b**) Gate charge characteristics of the conventional DTMOS and the proposed SiC MOSFET.

A comparison of the capacitance characteristics of the two studied SiC MOSFETs is shown in Figure 11. The input capacitance ($C_{\mathrm{iss}}$) and output capacitance ($C_{\mathrm{oss}}$) were nearly identical for both devices. The feedback capacitance ($C_{\mathrm{rss}}$ or $C_{\mathrm{GD}}$), however, was slightly larger for the proposed MOSFET than for the conventional DTMOS. Figure 12b shows the gate charge characteristics of the conventional DTMOS and the proposed SiC MOSFET. The gate–drain charge ($Q_{\mathrm{gd}}$) was observed within the plateau region of the gate charge curve. The extracted $Q_{\mathrm{gd}}$ values were 327 nC/cm^2^ for the conventional DTMOS and 346 nC/cm^2^ for the proposed SiC MOSFET. An increase in $Q_{\mathrm{gd}}$, caused by parasitic capacitance between the gate and drain, resulted in extended switching times. Although the $Q_{\mathrm{gd}}$ of the proposed structure increased slightly by 5.8%, its $R_{\mathrm{on},\mathrm{sp}}$ was reduced. As a result, the high-frequency figure of merit (HF-FOM), defined as $R_{\mathrm{on},\mathrm{sp}}\times Q_{\mathrm{gd}}$, was calculated to be 466.6 mΩ·nC for the conventional DTMOS and 418.3 mΩ·nC for the proposed structure. This represents a 10.4% reduction in the HF-FOM for the proposed structure compared to the conventional DTMOS, indicating its improved performance in high-frequency applications.

**Figure 13:**
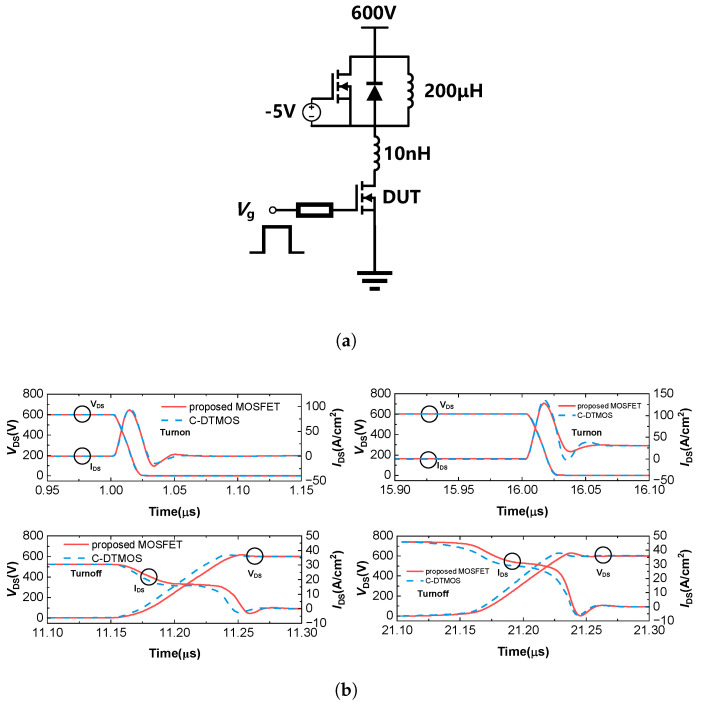
(**a**) Double-pulse test circuit of switching simulation. (**b**) Switching characteristics of the conventional DTMOS and the proposed SiC MOSFET.

**Figure 14:**
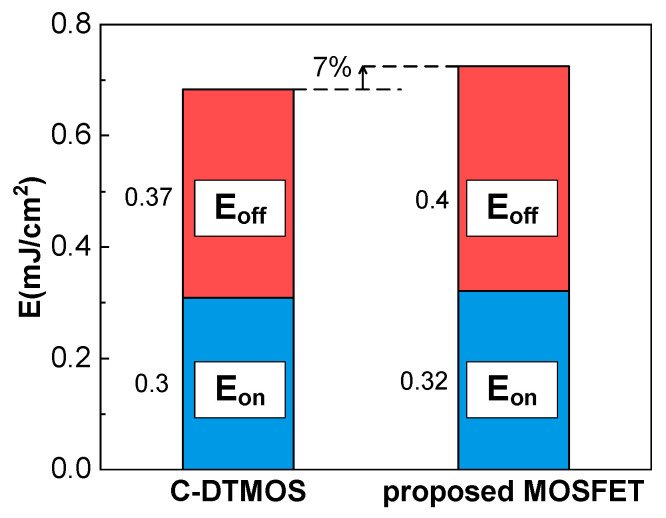
Comparison of the switching loss of the C-DTMOS and the proposed SiC MOSFET.

The switching characteristics of the conventional DTMOS and the proposed SiC MOSFET were evaluated using the double-pulse test circuit illustrated in Figure 13a. The switching waveforms are presented in Figure 13b, while Figure 14 provides a comparative analysis of the switching losses for both devices. It can be observed that the current and voltage waveforms during the first turn-on and turn-off were almost identical for the conventional DTMOS and the proposed structure. As a result, the turn-on and turn-off losses were nearly the same for both devices. However, due to the slightly higher $Q_{\mathrm{gd}}$ of the proposed structure compared to the conventional DTMOS, the total switching loss increased by 7% (from 0.67 to 0.72 mJ/cm^2^). However, during the second turn-on, it can be observed that the drain current ($I_{DS}$) of the proposed MOSFET settled oscillations more quickly than that of the conventional DTMOS. This is because the proposed MOSFET integrates a MOS-channel diode, which provides a faster reverse recovery speed, resulting in a quicker current recovery compared to the conventional DTMOS.

Table 2 presents a comparison of the key characteristics between the conventional DTMOS and the proposed SiC MOSFET. The proposed SiC MOSFET demonstrated improved performance, mainly due to the integration of the MCD structure. The reduced size of the bottom p-well in the proposed MOSFET resulted in a smaller depletion region, which, in turn, increased the current-carrying region when the device was in operation. This design effectively led to a significant reduction in $R_{\mathrm{on},\mathrm{sp}}$. Moreover, the threshold voltage ($V_{\mathrm{th}}$) of the device was determined by the size of the depletion region and the current flowing through the device.

## 4. Conclusions

In this article, a novel SiC double-trench MOSFET with an integrated MCD is proposed and simulated in Sentaurus TCAD. Due to the drain-induced barrier-lowering (DIBL) effect, the electron potential barrier is reduced by decreasing $L_{\mathrm{Ch}}$. As the channel length shortens, the barrier that electrons must overcome to flow from the source to the drain is significantly lowered, which, in turn, reduces the cut-in voltage ($V_{\mathrm{on}}$) and prevents current from flowing through the body diode. This effectively eliminates the risk of bipolar degradation. Furthermore, the breakdown voltage (BV) remains almost unchanged, while the switching loss only increases to 0.67 mJ/cm^2^ for the conventional DTMOS and to 0.72 mJ/cm^2^ for the proposed SiC MOSFET. The proposed structure not only eliminates bipolar degradation but also exhibits superior reverse conduction performance with minimal impact on other device characteristics. These advantages position the proposed SiC MOSFET as a strong competitor for power electronic applications.

## Figures and Tables

**Table 1:** Device parameters used in simulations.

Device Parameter	DTMOS	Proposed SiC MOSFET
Half-cell pitch	3 μm	3 μm
Drift thickness	5 μm	5 μm
Drift doping concentration	7 × 10^15^ cm^−3^	7 × 10^15^ cm^−3^
Trench width	1 μm	1 μm
Trench depth	1.5 μm	1.5 μm
P-well doping concentration	1 × 10^19^ cm^−3^	1 × 10^19^ cm^−3^
Gate oxide thickness	50 nm	50 nm
P-base width	1 μm	1 μm
P-base depth	0.5 μm	0.5 μm
P-base doping concentration	1 × 10^17^ cm^−3^	1 × 10^17^ cm^−3^
N+ doping concentration	1 × 10^19^ cm^−3^	1 × 10^19^ cm^−3^
P+ doping concentration	-	4 × 10^18^ cm^−3^
P-type contact resistance	1 × 10 ^−6^ Ω · cm^2^	1 × 10 ^−6^ Ω · cm^2^ [29]

**Table 2:** Comparison of device characteristics.

Parameter	DTMOS	Proposed SiC MOSFET
$V_{\mathrm{cut}-\mathrm{in}}$	2.57 V	0.83 V
$R_{\mathrm{on},\mathrm{sp}}$	1.427 mΩ·cm^2^	1.209 mΩ·cm^2^
BV	1014 V	997 V
$V_{\mathrm{th}}$	2.30 V	2.09 V
$Q_{\mathrm{gd}}$	327 nC/cm^2^	346 nC/cm^2^
$R_{\mathrm{on},\mathrm{sp}}\times Q_{\mathrm{gd}}$	466.6 mΩ·nC	418.3 mΩ·nC
$E_{\mathrm{on}}$	0.3 mJ/cm^2^	0.32 mJ/cm^2^
$E_{\mathrm{off}}$	0.37 mJ/cm^2^	0.4 mJ/cm^2^

## Data Availability

Data are contained within the article.
